# Prevalence and determinants of malaria infection among pregnant women attending antenatal clinic in Ejisu government hospital in Ghana: A cross-sectional study

**DOI:** 10.1371/journal.pone.0293420

**Published:** 2023-10-30

**Authors:** Catherine Kroamah Dwumfour, Victoria Bubunyo Bam, Lydia Boampong Owusu, Collins Atta Poku, Rhoda Dewe Kpabitey, Priscilla Aboagye, Amshariatu Suntaa Ibrahim

**Affiliations:** 1 Faculty of Allied Health Sciences, Department of Nursing, Kwame Nkrumah University of Science and Technology, Kumasi Ghana; 2 Maamobi General Hospital, Maternity Unit, Accra, Ghana; 3 Psychiatric Department, Suntreso Government Hospital, Kumasi, Ghana; University of Health and Allied Sciences, GHANA

## Abstract

**Introduction:**

Malaria in pregnancy is a global public health problem with the majority of its impact seen in sub-Saharan Africa. Pregnant women with malaria infection are at risk of adverse maternal outcomes. In Ghana, malaria in pregnancy accounts for about 17.6% of outpatient department attendance. Ashanti region is among the three regions with the highest malaria prevalence in pregnancy, particularly in the Ejisu Municipality. The study, therefore, assessed the prevalence and determinants of malaria infection among pregnant women seeking antenatal care at the Ejisu Government Hospital in Ghana.

**Methods:**

A cross-sectional study design with a convenience sampling technique was used to select 140 respondents for the study. Primary data such as age and residence of respondents were collected using a questionnaire and secondary data such as gestational age and Sulphadoxine Pyrimethamine (SP) administration were collected from clients’ maternal health record booklet. Bivariate and multivariate logistic regression analysis were used to assess the association between the malaria infection and the independent variables, and a p-value of < 0.05 was considered statistically significant.

**Results:**

The overall prevalence of malaria in pregnancy was 24 (17.1%). Most of the respondents had received counselling and health education 126 (90%), two or more doses of SP 95 (87.2%), Insecticide Treated Net (ITN) 99 (70.7%) and were sleeping under ITN 104 (74.3%). Multivariate logistic regression analysis showed a statistically significant association between malaria infection and sleeping under ITN (AOR = 0.05; 95% CI = 0.01–0.28, p< .001), the use of insecticide mosquito spray (AOR = 0.27; 95% CI = 0.09–0.84, p = .045) and reason for not using ITN due to the use of other preventive measures (AOR = 0.06; 95% CI = 0.01–0.61, p = .017).

**Conclusion:**

There was a high prevalence of malaria infection among study respondents despite the high usage of preventive measures for malaria in this study. It is therefore crucial that stakeholders in malaria control identify effective strategies to curb malaria transmission globally.

## Introduction

Malaria is a major public health burden worldwide with about 247 million cases of malaria and 619,000 malaria-related deaths occurring in 2021, globally [1]. In the same year, about 95% of world malaria cases and 96% of malaria-related deaths occurred in the WHO African Region [1].

Pregnant women and young children are the most vulnerable to malaria infections [2]. Global estimates indicate that in 2020, 121.9 million pregnancies were threatened by malaria infection in endemic areas [3]. In 2018, Sub-Saharan Africa (SSA) recorded about 11 million malaria infections in pregnant women [4]. West Africa alone recorded 6.5 million cases of malaria in pregnancy (MiP), the highest prevalence of exposure to malaria infection recorded in the WHO African region in 2021 [1].

Arguably, malaria infection is one of the greatest causes of maternal and foetal mortalities in SSA. It is associated with maternal anaemia, threatened abortion, intrauterine growth retardation, prematurity, neonatal and infant mortality and low birth weight [4–6]. In 2018, about 900,000 children in SSA were born with low birth weight [4]. In Mali, according to Mahamar et al. [7], malaria infection accounts for 3.5% of miscarriages, 3.2% of stillbirths, 2.8% of early neonatal death, 11.5% of preterm delivery, and 14.2% of small for gestational age among primigravid women. They also reported a lower rate of adverse maternal and foetal outcomes among secundigravidae and multigravidae. In Ghana, it accounts for 17.6% of outpatient department attendance [8].

Although literature exists regarding the prevalence and associated factors of malaria infection among pregnant women, data vary from setting to setting. A study conducted in southern Laos [9] and Ethiopia [10] recorded malaria infection prevalence of about 5.9% and 10.2% respectively as compared to 52.5% in Ondo State, Nigeria [11]. Ghanaian studies have reported a malaria infection prevalence rate of 14.1% among pregnant women in Northern [12] and 8.9% in a multicentre study conducted in middle and Southern Ghana [6]. Factors such as gravidity, maternal age and gestational age have been revealed as associated risk factors for malaria infection among pregnant women in Ghana [6,8,13]. Whereas studies conducted in Ethiopia [14] and Ghana [6] revealed that primigravidae were at the highest risk of malaria infections, another Ethiopian study by Gontie et al. [10] observed that secundigravidae were at the highest risk of malaria infection and a Malawian study [15] observed no association between gravidity or parity and malaria infection.

Given the dire health consequences associated with malaria in pregnancy and the threat it poses to both the pregnant mother and the foetus, the WHO recommended the use of insecticide-treated bed nets (ITNs), intermittent preventive treatment (IPT) with Sulfadoxine Pyrimethamine (SP), and prompt diagnosis and treatment of infection in pregnant women in malaria-endemic regions [1]. ITNs and IPT are effective in reducing malaria in pregnancy [16,17].

Since Ghana’s population is at a higher risk of malaria infection, several initiatives have been adopted to curb the situation [18,19]. Over the years, Global and national initiatives such as the Roll Back Malaria (RBM) partnership and Insecticide Resistance Monitoring (IRM) have been implemented to control the impact of malaria on vulnerable groups such as pregnant women and children under 5 years [19]. Following these initiatives, there was a significant decline in malaria parasite prevalence falling from 27.5% in 2011 to 14% in 2019 [20]. Despite this sharp decline, Ghana is still among the 15 malaria-burden countries in the world accounting for 3% of all global malaria infections and deaths [4].

ITN and IPTp are key preventive interventions pursued by Ghana to control malaria infection in pregnant women [21,22]. A systematic review of 5 randomized controlled trials by Gamble et al. [23] suggests that ITNs reduce malaria-related anaemia by 47%, low birth weight by 23%, miscarriages/stillbirths by 33% and placental parasitaemia by 23% [23]. A Cameroonian study showed that combined use of IPT and ITN was less associated with malaria infection than using IPT-SP or ITN alone [17]. Uptake of ≥3 doses of IPTp-SP is known to lower risks of placental malaria infection, preterm delivery and low birthweight in Ghana [24,25]. This shows that if IPTs-SP and ITNs are effectively used, malaria morbidity and mortality during pregnancy will reduce drastically. However, IPTs-SP are usually provided from the second trimester of pregnancy onwards because of the contraindication of SP during the first trimester due to its potential teratogenicity when used in the first trimester [26]. Therefore, pregnant women may be insufficiently or not protected during the first trimester of pregnancy. While ITN is routinely given to pregnant women in Ghana on their first ANC visit, recent evidence in Ghana suggests that the use of ITN and IPTp-SP uptake remains low [27,28]. The 2014–2019 Ghana Malaria Indicator Trends revealed a slight change in ITN use among pregnant women. The percentage of pregnant women who slept under an ITN the night before the survey ranged between 43% to 50% for the period under review. Despite a significant increase in women who received three or more doses of IPT-SP in the same period, IPT use remained unchanged between 2016 and 2019 [21]. According to the 2022 Ghana demographic and health survey [29], there was a low uptake of three or more doses of SP among women in their reproductive ages with a live birth in the 2 years preceding the survey. Ashanti region recorded 49.9% uptake of three or more doses of SP, the second lowest in the country and 36.9% use of ITNs among pregnant women aged 15–49 in all households that slept under an ITN the night before the survey, the third lowest in Ghana. The region also recorded the highest malaria admission rate in 2016 [22]. The 2022 Ejisu Municipal Assembly report indicated that the Ejisu Municipality had a high prevalence of malaria infection in the Ashanti region of Ghana [30]. Given this, there is the need to intensify efforts aimed at reducing malaria infections among pregnant women and this would require the availability of accurate and reliable data on the prevalence and associated factors of malaria across all parts of the world.

To the best of our knowledge, there is no current data on malaria prevalence and associated factors among pregnant women in the Ejisu Municipality. Identifying the associated factors of malaria infection in pregnant women will serve as indicators of susceptibility and risks of pregnant women to malaria. The findings of the current study will contribute to policy formulation and the progress of prevention campaigns to ensure an improvement in the healthcare of pregnant women. The study, therefore, assessed the prevalence and determinants of malaria infection among pregnant women seeking antenatal care at the Ejisu Government Hospital in Ghana.

## Methods

### Study design and setting

A cross-sectional study was conducted from 3^rd^ October to 4^th^ November 2022 at the Antenatal Clinic of the Ejisu Government Hospital in the Ashanti region of Ghana. Ejisu Government Hospital is a public health facility centrally located at Ejisu, the administrative capital of the Ejisu Municipal Assembly in the Ashanti Region of Ghana. It is a peri-urban community that serves as the major referral centre within the municipality. The municipality has a population of 180,723 according to the 2021 Population and Housing [31]. In 2017, the total skilled birth in the Ejisu Sub-municipality was 3872 [32]. There is a high prevalence of malaria in the Ejisu Municipality [30]. The Municipality recorded 18,335 malaria cases as of August 2022 [31]. Ejisu is located in the rainforest zone and its climatic conditions are typical of that of a tropical region and so aid in the transmission of malaria parasites (MPs) [23].

### Study population

The study population consisted of all pregnant women of reproductive ages (15 to 45 years) who attended antenatal care at the Ejisu Government Hospital during the study period. Pregnant women with a record of malaria test at first or current visit were included in the study. Those who were coming for ANC for the first time were excluded from the study as they had not yet been tested for malaria parasites.

### Sampling and sample size determination

Pregnant women who were eligible for the study were recruited through convenience sampling. The sample size calculation was based on the Cochran 1963 sample size formula ($\text{n}=\frac{z^{2}(p)(q)}{{\left(e\right)}^{2}}$) [33], with a 95% confidence interval, 5% acceptable sample error and malaria prevalence of 10% among pregnant women. The minimum sample size required for the study was calculated as 138. A final sample size of 152 respondents was used after adding a 10% dropout rate [34,35].

### Variable measurement and data collection tool

The dependent variable for this study was malaria infection which was assessed with RDT and microscopy results. Malaria infection was defined as either a positive RDT or a positive microscopy result. The independent variables include; sociodemographic characteristics of respondents (age, religion, marital status, residence, occupation, level of education, and monthly income); obstetric factors (gestational age, gravidity, parity, attended all ANC visits, number of ANC visits, and gestational age at first ANC visit); preventive measures of malaria (counselling and health education, forms of education received, SP intake, doses of SP, received ITN, sleeps under ITN, gestation at which respondent started sleeping under ITN, type of ITN used, source of ITN, use of mosquito repellent, insecticide spray, mosquito coil, fan and anti-mosquito LED light).

Data was collected using a self-administered questionnaire. The researchers developed the questionnaire based on the study objectives and an extensive literature review of the variables [5,6,8,10]. Data on sociodemographic data, obstetric history, prevalence of malaria infection, and utilization of malaria preventive measures among pregnant women were collected using the questionnaire. The questionnaire was pre-tested among 5% of the study sample at the Living Waters hospital at Ejisu to ensure reliability, and it yielded a Cronbach alpha of 0.85.

### Data collection process

Both primary and secondary data were collected on the respondents. Primary data such as age and ITN use by respondents were collected using the questionnaire. The following secondary data were extracted from the ANC booklet of the respondents; number of ANC visits, gravidity, parity and gestational age, and malaria test results recorded during the first and current ANC and administration of SP and ITN. All respondents were informed about the purpose of the study, and those who consented to participate in the study signed a written consent form. The questionnaire, which was prepared in English Language was given to the respondents to answer. For respondents who did not understand the English language, diligent efforts were made by the researchers to explain the questionnaire in the local language (“Twi”). All researchers were trained to administer the questionnaire in both English and Twi, having ensured that there was uniformity in the explanation of questions and processes. Nonetheless, only one of the researchers collected data on respondents who could not understand English.

### Data analysis

Data analysis was performed using Statistical Package for Social Sciences (SPSS) version 25. Descriptive statistics was used in the analysis of socio-demographic characteristics, obstetric history, prevalence of malaria infection and preventive measures of malaria infection and variables were presented as frequencies and percentages. Bivariate and multivariate logistic regression analysis were performed to determine the association between malaria infection and the independent categorical data, and a p-value of < 0.05 was considered statistically significant.

## Results

### Sociodemographic characteristics of respondents

One hundred and forty (140) questionnaires were retrieved and used for the data analysis giving a response rate of 92.1%. The socio-demographic characteristics of respondents are presented in Table 1. About 59 (42.1%) of the respondents were within the age range of 25–34 years. The majority of the respondents 88 (62.9%) were Christians. More than half 88 (62.9%) of the respondents were married and more than two-thirds 99 (70.7%) were urban residents. About half 71 (50.7%) of the respondents were self-employed and a little below a third 40 (28.6%) of the respondents were unemployed. Two-fifths 57 (40.7%) of respondents had Senior High School education. More than two-thirds 98 (70%) of the respondents earned less than $50 a month.

**Table 1:** Socio-demographic characteristics of respondents.

Variables	Frequency (%)
**Age**	
15–24	50 (35.7)
25–34	59 (42.1)
35–45	31 (22.1)
**Religion**	
Christian	88 (62.9)
Muslim	51 (36.4)
Traditionalist	1 (0.7)
**Marital status**	
Single	40 (28.6)
Married	88 (62.9)
Divorced	2 (1.4)
Cohabiting	10 (7.1)
**Residence**	
Urban	99 (70.7)
Rural	41 (29.3)
**Occupation**	
Employed (Government and Private)	29 (20.7)
Self-employed	71 (50.7)
Unemployed	40 (28.6)
**Level of education**	
No formal education	6 (4.3)
Primary	6 (4.3)
Junior high school	51 (36.4)
Senior secondary school	57 (40.7)
Tertiary education	20 (14.3)
**Monthly income** Less than $50	98 (70)
$50 ‐ $100	34 (24.3)
Above $100	8 (5.7)

### Obstetric history of the respondents

The obstetric history of respondents is illustrated in Table 2. The majority of the respondents 72 (51.4%) were in their second trimester of gestation. More than half 49 (35%) of the respondents were gravida two. About a third 45 (33.6%) and 47 (32.1%) of the respondents were para 0 and 1 respectively. The majority 134 (95.7%) of the respondents attended all scheduled ANC visits and more than half 83 (59.3%) have had at least 4 or more ANC visits during the current pregnancy. Most 88 (62.9%) of the respondents had their first ANC visit within the first trimester.

**Table 2:** Obstetric history of respondents.

Variable	Frequency (%)
**Gestational age (weeks)**	
≤12 weeks	20 (14.3)
13–28 weeks	72 (51.4)
29–40 weeks	48 (34.3)
**Gravidity**	
1	42 (30.0)
2	49 (35.0)
3	27 (19.3)
4	15 (10.7)
≥5	7 (5)
**Parity**	
0	47 (33.6)
1	45 (32.1)
2	27 (19.3)
3	15 (10.7)
4	5 (3.6)
≥5	1 (0.7)
**Attended all ANC**	
Yes	134 (95.7)
No	6 (4.3)
**Number of ANC visits**	
< four times	57 (40.7)
≥ four times	83 (59.3)
**Gestational age at 1**^**st**^ **visit**	
≤ 12 weeks	88 (62.9)
13–28 weeks	50 (35.7)
29–40 weeks	2 (1.4)

### Prevalence of malaria infection

The prevalence of malaria infection among the respondents is detailed in Table 3. All the respondents had malaria tests done during the current pregnancy. Less than a fifth, 24 (17.1%) of the respondents tested positive for malaria parasites. Out of the 24 respondents who tested positive for malaria parasites, the majority 21 (87.5%) of them had one episode of malaria infection and less than a fifth 3 (12.5%) had two episodes of malaria infection in their current pregnancy. Blood film for MPs was the most common diagnostic test used for respondents 20 (83.3%). A third, 9 (37.5%) of the respondents indicated that there were breeding sites for malaria infection in their area of residence.

**Table 3:** Prevalence of malaria infection among respondents.

Variable	Frequency (%)
**Tested positive for malaria**	
Yes	24 (17.1)
No	116 (82.9)
**Episodes of malaria**	
One	21 (87.5)
Two	3 (12.5)
**Diagnostic test used**	
RDT	4 (16.7)
Blood film or MPs	20 (83.3)
**Are there any mosquito breeding sites** **in your area of residence?**	
Yes	9 (37.5)
No	15 (62.5)

### Preventive measures of malaria infection

Table 4 details the preventive measures for malaria infection. The majority 126 (90%) of the study respondents received counselling and health education on malaria infection. Group education was the most common type of education given, 82 accounting for 65.1% of the respondents. More than two-thirds 109 (77.9%) of respondents received SP. Most 80 (73.4%) of the respondents had received two to three doses of SP. Out of the 31 (22.1%) respondents who did not receive SP, 25 (80.6%) of them did not take SP because they were in the first trimester of pregnancy. More than two-thirds 99 (70.7%) of respondents received ITN from the facility. The majority 104 (74.3%) of respondents sleep under ITN. More than half 58 (55.8%) of respondents always slept under ITN. The most common reason for not using ITN was not having ITN 17 (47.2%). Out of the 104 (74.3%) respondents who slept under ITN, about half 54 (51.9%) of them started sleeping under ITN in their first trimester. More than four-fifths, 93 (89.4%) of the respondents used long-lasting insecticide nets (LLINs). The majority 104 (74.3%) of respondents received ITN from healthcare professionals. About 68 (48.6%) of respondents use mosquito repellent and spray. Less than half 61 (43.6%) of the respondents kept their environment clean, one-fourth 4 (3.5% of the respondents used mosquito coils and about 5 (3.6%) used anti-mosquito led light to prevent malaria.

**Table 4:** Preventive measures of malaria infection.

Preventive measure	Frequency (%)
**Counselling and health education**	
Yes	126 (90.0)
No	14 (10.0)
**Form of education received**	
Individual	12 (9.5)
Group	82 (65.1)
Both	32 (25.4)
**Received SP**	
Yes	109 (77.9)
No	31 (22.1)
**Doses of SP**	
One	14 (12.8)
Two	33 (30.3)
Three	47 (43.1)
More than three	15 (13.8)
**Reason for not taking SP n = 31**	
Unaware of SP	1(3.2)
Positive for G6PD	5 (16.1)
First trimester of gestation	25(80.6)
**Received ITN**	
Yes	99 (70.7)
No	41 (29.3)
**Sleep under ITN**	
Yes	104 (74.3)
No	36 (25.7)
**How often do you sleep under ITN (n = 104)**	
Always	58 (55.8)
Sometimes	42 (40.4)
Rarely	4 (3.8)
**Reasons for not sleeping under ITN**	
do not have	17 (47.2)
Uncomfortable	6 (16.7)
first ANC visit	1 (2.7)
use of other preventive measures	12 (33.3)
**Gestation started sleeping under ITN**	
≤12 weeks	54 (51.9)
13–28 weeks	47 (45.2)
29–40 weeks	3 (2.9)
**Type of ITN used**	
ordinary bed net	11 (10.6)
LLINs	93 (89.4)
**Source of ITN**	
Healthcare provider	104 (74.3)
Previously owned	5 (3.6)
Purchased from market	4 (2.9)
Gift	27 (19.3)
**Use of mosquito repellent**	
Yes	68 (48.6)
No	72 (51.4)
**Use of insecticide mosquito spray**	
Yes	68 (48.6)
No	72 (51.4)
**Keeping the environment clean**	
Yes	61 (43.6)
No	79 (56.4)
**Use of mosquito coil**	
Yes	35 (25.0)
No	105 (75.0)
**Use of fan**	
Yes	4 (2.9)
No	136 (97.1)
**Use of anti-mosquito LED light**	
Yes	5 (3.6)
No	13 (96.4)

SP: Sulfadoxine pyrimethamine; LLINs: Long-lasting insecticide nets; ITN: Insecticide Treated Nets.

### Factors associated with malaria infection

Table 5 presents the factors associated with malaria infection among respondents. Out of the 30 independent variables of interest, only 5 were significant in the bivariate regression model while 3 of them were significant in the multivariate regression model at the 95% C.I. A multivariate logistic regression analysis showed that the chances of being infected with malaria is reduced by 95% in pregnant women who sleep under ITN (p< 0.001). Again, the chances of being infected with malaria are reduced by 94% in respondents whose reason for not using ITN was due to the use of other preventive measures (p = 0.017). In addition, the chances of being infected with malaria are reduced by 73% in respondents who use insecticide mosquito spray as an alternative to the use of ITN (p = .045).

**Table 5:** Factors associated with malaria infection among respondents.

Factor	Bivariate		Multivariate	
	COR (95% C.I.)	p-value	AOR (95% C.I.)	p-value
**Gestational age at 1**^**st**^ **visit**		.021		
13–28 weeks	0.54 (0.16–1.79)		0.30 (0.08–1.05)	.050
**Sleeping under ITN**		.018		
Yes	0.32 (0.13–0.81)		0.05 (0.01–0.28)	**< .001**
**How often do participants sleep under ITN?**		.024		
Sometimes	3.18 (0.89–11.36)		3.45 (0.86–13.86)	.140
Rarely	4.5 (0.38–53.77)		5.91 (0.35–100.57)	.257
**Reason for not sleeping under ITN**		.003		
Uncomfortable	0.18 (0.02–1.86)		0.26 (0.02–3.30)	.287
Use of other preventive measures	0.08 (0.01–0.39)		0.06 (0.01–0.61)	**.017**
**Use of insecticide mosquito spray**		.048		
Yes	0.38 (0.15–0.97)		0.27 (0.09–0.84)	**.045**

COR: Crude Odds Ratio; AOR: Adjusted Odds Ratio; C.I.: Confidence Interval.

## Discussion

Malaria infection is endemic among people living in tropical regions and it is associated with morbidities and mortalities, especially among vulnerable populations like pregnant women [8]. The study was therefore carried out to assess the prevalence and risk factors of malaria infection among pregnant women at the Ejisu Government Hospital situated in a community with high malaria transmission.

The prevalence of malaria infection among the respondents in this study was 17.1% which is similar to the prevalence of malaria among pregnant women in Ghana [8]. The figure is also comparable to findings of similar studies conducted in Ethiopia [36] and Burkina Faso [37], but higher than studies conducted in Southern Laos [9], Ghana [6,13] and Sudan [38]. The prevalence is, however, lower than that reported among pregnant women in Hohoe, Ghana [39], and in Ondo State, Nigeria [11]. These variations could be attributed to the difference in geographical area, period of data collection, sample size, adherence to malaria preventive measures and the type of diagnostic test done. Again, the use of either RDT or microscopy diagnostic tests in the present study might have contributed to the higher prevalence recorded in the study. Although microscopy is the gold standard for malaria diagnostic tests, RDT and microscopy are the two common tests for malaria in Ghana [40]. Hence using positive microscopy results alone which was done by Fonjo et al. [6] study implies that respondents who tested positive for malaria parasite with RDT would have been missed reducing the prevalence of malaria in the study.

The present study was conducted between October to November and was characterized by minor rainfall, humidity, and warmth [30]. Adequate rainfall, humidity, and warmth facilitate the breeding of mosquitoes and the transmission of malaria [41]. Moreover, this study was conducted in a peri-urban community. Evidence suggests that peri-urban communities have a lower and higher prevalence of malaria compared to rural and urban communities respectively [42]. The high prevalence in our study could have also been influenced by the fact that more than a third (37.5%) of the respondents confirmed that there were breeding sites of mosquitoes in their area and about one-fourth of the respondents were not sleeping under ITN. Thus, a multipronged approach is needed to curb malaria transmission. There is a need for stakeholders such as the Family Health Division of the Ghana Health Service to reinforce community engagement in the control and prevention of malaria. Community Health Planning Services (CHPs), political, private sector, traditional leaders, faith-based leaders and community volunteers have a key role to play in community-based interventions which include behavioural changes, surveillance and service provision [43]. Community-based health education, regular cleaning of the environment, clearing of bushes, and draining of stagnant waters should be encouraged by the local government to control and prevent malaria. When members of the community are involved in interventions and policy formulation, they are more likely to adhere to preventive strategies [43] which will help reduce malaria in endemic areas. Barry et al. [44] found a link between head of household and malaria preventive measures among pregnant women in Guinea. A Ghanaian study found that homes that were headed by males were more likely to own ITN [45]. As a result, health workers should strengthen partner involvement in ANC activities as this can help drive home preventive education on malaria, such as ITN use. The 2022 World Malaria Report revealed a decline in funding for malaria prevention and control interventions since 2019. This decline can lead to an increase in malaria cases, malaria-associated deaths and economic loss to the country. Thus, sustained malaria financing is crucial for malaria elimination in the country. The government of Ghana should therefore increase domestic funding while non-governmental agencies such as the Bill and Melinda Gates Foundation also increase global funding for the prevention and control of malaria infection [46]. Since the high prevalence of malaria can lead to dire consequences, the Ministry of Health (MoH), Ghana Health Service (GHS) and National Malaria Control Programme (NMCP) should employ rigorous strategies to reduce malaria parasites by conducting high-quality research and implementing current research findings on malaria prevention [47] such as exploring the impact of malaria antibodies vaccination in pregnant women [48] and also explicitly stating in guidelines/reports how to implement and monitor Directly Observed Therapy (DOT) of IPT [49]. The requirements for implementation of DOT, according to Al Khaja and Sequeira [49] have not been explicitly stated in national guidelines/reports in Ghana and this has made its implementation in real-world practice uncertain leading to poor adherence. This finding is supported by Dun-Dery et al. [28] study where the international recommendation of folic acid dose administration with SP was contrary to what was done in many countries.

The WHO recommend sleeping under ITN and indoor residual spraying (IRS) to prevent mosquito bites or chemoprevention (IPTp-SP) to suppress malaria infections in sub-Saharan Africa where malaria is endemic (3). The study found a significant association between malaria infection and sleeping under ITN. The chances of being infected with malaria are reduced by 95% in pregnant women who sleep under ITN (p < 0.001). The study findings are consistent with studies conducted in Ethiopia [10], Ghana [13] and Eastern Indonesia [50] where the use of insecticide-treated nets was significantly associated with reduced odds of malaria infection.

Contrarily, some studies [5,6,11,37] found no significant association between malaria and ITN use. This may be because most of the pregnant women in our study used ITN mainly to control mosquito bites. About 74.3% of the respondents in our study slept under ITN as compared to the 53% recorded by WHO among pregnant women in SSA in 2021 [1] and 58% recorded in Ghana in 2019 [21]. The use of ITN was much closer to the 80% target by WHO [51] and NMCP [21]. This may have accounted for the significant association between malaria and ITN use in our study. The high uptake of ITN especially, LLINs among respondents may be attributed to the strategies put in place by the WHO and NMCP of Ghana to protect about 80% of eligible pregnant women from malaria infection. In Ghana, pregnant women are given free ITNs on their first ANC visit [21,22]. This policy may have also contributed to the high usage of ITN in our study.

Despite this success, about 29.3% of pregnant women in our study did not receive ITN from the facility during their current pregnancy. This could be due to shortages of ITNs in the facility. The result is in line with studies conducted in the Volta and Ashanti regions in Ghana which reported that some women did not receive ITN in their initial and subsequent ANC visits due to health system challenges such as stock out secondary to transportation to district health directorate to collect ITN stocks [52,53]. Both studies found that even when stocks were available in the healthcare facility women who did not receive ITN in their first ANC visit were not given ITN [52,53]. District health directorates and health facilities should always provide transport to managers to convey ITN to health facilities even before stockout. ANC managers and Reproductive and Child Health Unit managers should also make prompt requisitions for ITN before stockout. All efforts should be made by ANC managers, Reproductive and Child Health Unit managers and midwives to routinely check clients’ maternal health records to identify women who have not received ITN in their first ANC attendance so that ITN is issued to them.

The chances of being infected with malaria are reduced by 94% in respondents whose reason for not using ITN was due to the use of other preventive measures (p = 0.017). The use of insecticide mosquito spray as an alternative to the use of ITN was statistically associated with malaria infection in this current study. Likewise, Ipa et al. [50] found an association between other measures of malaria prevention such as the use of mosquito repellents and coils. Pregnant women used other preventive measures such as insecticide mosquito spray and coils, keeping the environment clean, fans and antimalaria LED light. A study conducted in Ghana among the rural communities in Ho reported similar findings [54]. On the contrary, a review of two randomized controlled trials and eight cluster-randomized trials reported inconclusive results on the use of malaria repellents both topical and spatial as a malaria preventive tool due to the low certainty evidence [55]. However, pregnant women should use these interventions rather than not use any preventive measures for malaria. There is therefore the need for population-based studies on alternative methods of malaria control and prevention using vigorous methodology. Health workers should continuously emphasize the benefits of recommended malaria preventive measures such as ITNs and SP at ANC visits. Health education is critical in the control of malaria among pregnant women because it promotes the use of malaria-preventive strategies [10]. In our study, only a few of the client received individual education during their ANC attendance. Sunuwar et al. [56], found a higher knowledge score and improved dietary intake of iron-rich foods among pregnant women who received individual education during antenatal care. Antenatal care allows healthcare providers to give preventive services to pregnant women. Hence, health education should be tailored to the individual needs of pregnant women at ANC visits to promote uptake of preventive measures.

### Limitation

A cross-sectional study design was used therefore, the study could not demonstrate a causal relationship between the outcome and the explanatory variables. Some aspects of the data collection included self-reported data which introduces reporting bias. The use of the convenience sampling method also introduced selection bias. The small sample size could limit the generalization of the results and also impact the accuracy of the study findings, therefore, findings should be generalized with caution.

## Conclusion

The prevalence of malaria among pregnant women at the Ejisu Government Hospital was high. Sleeping under ITN, use of insecticide mosquito spray and reason for not sleeping under ITN due to the use of other preventive measures were significantly associated with malaria infection. Therefore, health workers should give intensive education on the benefits of malaria preventive measures and early ANC care in the community and health facilities. They should also encourage women to use recommended malaria control measures and attend ANC care in their first trimesters so that they can be tested for malaria and treated early to improve outcomes for the mother and baby. In addition, governmental agencies including MOH and GHS and non-governmental agencies such as the Bill and Melinda Gates Foundation should take extra measures such as increasing funding for malaria control and prevention programmes to reduce the rate of infection in malaria-endemic areas.

## Supporting information

S1 Checklist STROBE statement—checklist of items that should be included in reports of observational studies. (DOCX)

## Abbreviations

ANC: Antenatal care
GHS: Ghana Health Service
GSS: Ghana Statistical Service
IPT: Intermittent preventive treatment
IPTp-SP: Intermittent preventive treatment of malaria in pregnancy with Sulfadoxine pyrimethamine
IRM: Insecticide resistance monitoring
IRS: Indoor residual spraying
ITN: Insecticide-treated net
LLINs: Long-lasting insecticide net
MiP: Malaria in pregnancy
NMCP: National Malaria Control Programme
MPs: Malaria parasites
RDT: Rapid diagnostic test
SP: Sulfadoxine pyrimethamine
RBM: Roll Back Malaria partnership
SSA: Sub-Saharan Africa
WHO: World Health Organization
