# Supplementary material for: Prevalence and determinants of malaria infection among pregnant women attending antenatal clinic in Ejisu government hospital in Ghana: A cross-sectional study
Source: PLoS One. 2023 Oct 30;18(10):e0293420. doi: 10.1371/journal.pone.0293420 (PMC10615274; doi:10.1371/journal.pone.0293420)
Supplement: S1 Checklist — (DOCX) [file pone.0293420.s001.docx]

STROBE Statement—checklist of items that should be included in reports of observational studies

|  | Item No. | Recommendation | Page  No. | Relevant text from manuscript |
| --- | --- | --- | --- | --- |
| **Title and abstract** | 1 | (*a*) Indicate the study’s design with a commonly used term in the title or the abstract | 2 | A cross sectional study was used. |
|  |  | (*b*) Provide in the abstract an informative and balanced summary of what was done and what was found | 2 | Cross-sectional design with a convenience sampling technique was used to select 140 respondents for the study. Primary data and Secondary data were collected from respondents. Binary logistic regression was used to assess the association between the malaria infection and the independent variables. The study found a 17.1% prevalence of malaria in pregnancy. Chi-square analysis showed a statistically significant associated between malaria infection and sleeping under ITN, reason for not sleeping under ITN, how often respondents sleep under ITN, source of ITN, the use of insecticide mosquito spray and use of mosquito led light. Bivariate analysis revealed an association between gestation at first ANC attendance and malaria infection (OR=3.43; 95%CL = 1.05-11.18, P=0.041). There was also a statistical association between not sleeping under ITN, do not have ITN and non-use of mosquito spray and malaria infection. |
| Introduction | | | |  |
| Background/rationale | 2 | Explain the scientific background and rationale for the investigation being reported | 3 | Malaria is a major public health burden worldwide affecting about 3.3 billion people of all ages. In 2021, about 95% of world malaria cases and 96% of malaria-related deaths occurred in the WHO African Region. Pregnant women are among the most vulnerable group of malaria infections. It threatens 17.6% of all pregnancies in  Ghana. Malaria is one of the greatest causes of maternal and foetal mortalities in SSA. It is associated with maternal anaemia, threatened abortion, intrauterine growth retardation, prematurity, neonatal and infant mortality and low birth weight. |
| Objectives | 3 | State specific objectives, including any prespecified hypotheses | 4 | The study assessed the prevalence and determinants of malaria infection among pregnant women seeking antenatal care at the Ejisu Government Hospital in Ghana. |
| Methods | | | |  |
| Study design | 4 | Present key elements of study design early in the paper | 5 | Cross-sectional study design |
| Setting | 5 | Describe the setting, locations, and relevant dates, including periods of recruitment, exposure, follow-up, and data collection | 5 | The study was conducted from October to November 2022 at the Antenatal Clinic of the Ejisu Government Hospital in the Ashanti region of Ghana. Ejisu Government Hospital is a public health facility centrally located at Ejisu, the administrative capital of the Ejisu Municipal Assembly in the Ashanti Region of Ghana-West Africa. It is a peri-urban community that serves as the major referral centre within the municipality. |
| Participants | 6 | (*a*) *Cohort study*—Give the eligibility criteria, and the sources and methods of selection of participants. Describe methods of follow-up  *Case-control study*—Give the eligibility criteria, and the sources and methods of case ascertainment and control selection. Give the rationale for the choice of cases and controls  *Cross-sectional study*—Give the eligibility criteria, and the sources and methods of selection of participants | 5 | N/A  Pregnant women aged 15 to 45 years who attends the antenatal clinic at the Ejisu Government Hospital during the study period. Pregnant women with record of malaria test at first or current visit were included in the study. Those who were coming for ANC for the first time were excluded from the study as they had not yet been tested for malaria parasite. Pregnant women who were eligible for the study were recruited through convenience sampling. |
|  |  | (*b*) *Cohort study*—For matched studies, give matching criteria and number of exposed and unexposed  *Case-control study*—For matched studies, give matching criteria and the number of controls per case |  | N/A  N/A |
| Variables | 7 | Clearly define all outcomes, exposures, predictors, potential confounders, and effect modifiers. Give diagnostic criteria, if applicable | 5-6 | Dependent variable: Malaria infection  Independent variables: sociodemographic characteristic of respondents (age, religion, marital status, residence, occupation, level of education, and monthly income); obstetric factors (gestational age, gravidity, parity, attended all ANC visits, number of ANC visits, and gestational age at first ANC visit); preventive measures of malaria (counselling and health education, forms of education received, SP intake, doses of SP, received ITN, sleeps under ITN, gestation at which respondent started sleeping under ITN, type of ITN used, source of ITN, use of mosquito repellent, insecticide spray, mosquito coil, fan and anti-mosquito led light). |
| Data sources/ measurement | 8* | For each variable of interest, give sources of data and details of methods of assessment (measurement). Describe comparability of assessment methods if there is more than one group | 6 | Researcher developed questionnaire based on the study objectives and extensive literature review was used to collect; sociodemographic data, obstetric history, prevalence of malaria infection, and utilization of malaria preventive measures among the pregnant women. The following data were also extracted from the ANC booklet of the pregnant women; number of ANC visits, gravidity, parity and gestational age, malaria test results recorded during the first and current ANC and administration of SP and ITN. |
| Bias | 9 | Describe any efforts to address potential sources of bias | 6 | Secondary data from respondents ANC booklet was used to reduce the bias |
| Study size | 10 | Explain how the study size was arrived at | 5 | The Cochran 1963 sample size formula (n= $\frac{z^{2}(p)(q)}{{(e)}^{2}}$), with 95% confidence interval and 5% acceptable sample error. The prevalence of malaria among pregnant women in middle and southern Ghana has been estimated by Fondjo et al., (2020) as 8.9%. We anticipated a malaria prevalence of 10% among pregnant women in the present study. |

Continued on next page

| Quantitative variables | 11 | Explain how quantitative variables were handled in the analyses. If applicable, describe which groupings were chosen and why | 6 | Frequencies and percentages were used to describe sociodemographic data, obstetric history and preventive measures of malaria infection. |
| --- | --- | --- | --- | --- |
| Statistical methods | 12 | (*a*) Describe all statistical methods, including those used to control for confounding | 6 | Statistical analysis was performed using Statistical Package for Social Sciences (SPSS) version 25. Descriptive statistics for categorical data were presented as frequencies and percentages. Chi-square test was performed to determine the association between malaria infection and the independent categorical data, followed by binary logistic regression analysis. P-value < 0.05 was considered statistically significant. |
|  |  | (*b*) Describe any methods used to examine subgroups and interactions |  | N/A |
|  |  | (*c*) Explain how missing data were addressed |  | There were no missing data |
|  |  | (*d*) *Cohort study*—If applicable, explain how loss to follow-up was addressed  *Case-control study*—If applicable, explain how matching of cases and controls was addressed  *Cross-sectional study*—If applicable, describe analytical methods taking account of sampling strategy |  | N/A  N/A  N/A |
|  |  | (*e*) Describe any sensitivity analyses |  | N/A |
| Results | | | | |
| Participants | 13* | (a) Report numbers of individuals at each stage of study—eg numbers potentially eligible, examined for eligibility, confirmed eligible, included in the study, completing follow-up, and analysed | 5,7 | 152 respondents were recruited for the study. 140 respondents who met the eligibility criteria were included in the data analysis. The response rate was 92.1%. |
|  |  | (b) Give reasons for non-participation at each stage |  | N/A |
|  |  | (c) Consider use of a flow diagram |  | N/A |
| Descriptive data | 14* | (a) Give characteristics of study participants (eg demographic, clinical, social) and information on exposures and potential confounders | 7-8 | Socio-demographic characteristics: Majority of the respondents were within the age range of 25-34 years, married, were Christians, lived in urban communities, were self-employed, had Senior High School education and earned less than $50 a month. |
|  |  | (b) Indicate number of participants with missing data for each variable of interest |  | N/A |
|  |  | (c) *Cohort study*—Summarise follow-up time (eg, average and total amount) |  | N/A |
| Outcome data | 15* | *Cohort study*—Report numbers of outcome events or summary measures over time |  | N/A |
|  |  | *Case-control study—*Report numbers in each exposure category, or summary measures of exposure |  | N/A |
|  |  | *Cross-sectional study—*Report numbers of outcome events or summary measures | 9-10 | Prevalence of malaria infection in pregnancy. |
| Main results | 16 | (*a*) Give unadjusted estimates and, if applicable, confounder-adjusted estimates and their precision (eg, 95% confidence interval). Make clear which confounders were adjusted for and why they were included | 12-14 | N/A  None of the variables were adjusted |
|  |  | (*b*) Report category boundaries when continuous variables were categorized |  | NA |
|  |  | (*c*) If relevant, consider translating estimates of relative risk into absolute risk for a meaningful time period |  | N/A |

Continued on next page

| Other analyses | 17 | Report other analyses done—eg analyses of subgroups and interactions, and sensitivity analyses |  | N/A |
| --- | --- | --- | --- | --- |
| Discussion | | | | |
| Key results | 18 | Summarise key results with reference to study objectives | 15-17 | The study assessed the prevalence and determinants of malaria infection among pregnant women seeking antenatal care at the Ejisu Government Hospital in Ghana.  The prevalence of malaria infection in this study was 17.1%.  Sleeping under ITN (p<0.05), reason for not sleeping under ITN (p<0.001), how often respondents sleep under ITN (p<0.05), source of ITN (p<0.001), the use of insecticide mosquito spray (p<0.05) and use of mosquito led light (p<0.001), first ANC attendance between 13 to 28 weeks gestation (OR=3.43; 95%CL = 1.05-11.18, p<0.05), receiving ITN from a friend (OR=27.42; 95%CL=1.20-629.03, p<0.05), not sleeping under ITN (OR=0.10; 95%CL = 0.01-0.45, p<0.05), do not have ITN (OR=0.02; 95%CL = 0.00-0.36, p<0.05) and non-use of mosquito spray (OR=0.32; 95%CL = 0.10-0.98, p<0.05) were the determinants of malaria infection. |
| Limitations | 19 | Discuss limitations of the study, taking into account sources of potential bias or imprecision. Discuss both direction and magnitude of any potential bias | 17 | A cross-sectional study design was used which could not demonstrate a causal relationship between the outcome and the explanatory variables. Some aspects of the data collection included self-reported data which introduces reporting bias however, the use of secondary data from ANC reduced the bias. The small sample size could have limited the power of the study which may have affected the results. The use of convenience sampling method also introduced selection bias therefore findings should be generalized with caution. |
| Interpretation | 20 | Give a cautious overall interpretation of results considering objectives, limitations, multiplicity of analyses, results from similar studies, and other relevant evidence | 17 | Overall, the prevalence of malaria among pregnant women at the Ejisu Government Hospital was high as compared to studies conducted in Nigeria, Ghana and Sudan. Ownership and use of ITN, how often respondents sleep under ITN, source of ITN, use of insecticide mosquito spray, mosquito led light and gestation at first ANC attendance were significantly associated with malaria infection. Similar findings have been reported in literature. |
| Generalisability | 21 | Discuss the generalisability (external validity) of the study results | 5 | Small sample size  Convenience sampling method Findings should be generalized with caution. The study should be generalized to settings with similar characteristics. |
| Other information | |  | | |
| Funding | 22 | Give the source of funding and the role of the funders for the present study and, if applicable, for the original study on which the present article is based | 18 | No funding was received for this study. |

*Give information separately for cases and controls in case-control studies and, if applicable, for exposed and unexposed groups in cohort and cross-sectional studies.

**Note:** An Explanation and Elaboration article discusses each checklist item and gives methodological background and published examples of transparent reporting. The STROBE checklist is best used in conjunction with this article (freely available on the Web sites of PLoS Medicine at http://www.plosmedicine.org/, Annals of Internal Medicine at http://www.annals.org/, and Epidemiology at http://www.epidem.com/). Information on the STROBE Initiative is available at www.strobe-statement.org.
